# Recurrent Nonobstructive Cholangitis After Pancreaticoduodenectomy: Prevalence, Risk Factors, and Treatment

**DOI:** 10.1002/wjs.70037

**Published:** 2025-08-07

**Authors:** Anaïs Choquet, Aurélien Sokal, Safi Dokmak, Audrey Le Bot, Fanny Delahaye, Jeanne Dembinski, Béatrice Aussilhou, Vinciane Rebours, Victoire de Lastours, Alain Sauvanet

**Affiliations:** ^1^ Department of HPB Surgery AP‐HP Beaujon Hospital University of Paris‐Cité Clichy France; ^2^ Department of Internal Medecine AP‐HP Beaujon Hospital University of Paris‐Cité Clichy France; ^3^ Department of Pancreatology AP‐HP Beaujon Hospital University of Paris‐Cité Clichy France

**Keywords:** cholangitis, IPMN, pancreaticoduodenectomy, postoperative complications

## Abstract

**Background:**

Recurrent nonobstructive cholangitis (RNOC) due to enterobiliary reflux is a late complication of pancreaticoduodenectomy (PD) that should be differentiated from obstructive cholangitis. The risk factors (RFs) of RNOC are poorly known. The goal of this study was to estimate the prevalence, to identify the RF, and to describe the management of RNOC following PD.

**Methods:**

This case–control study included 503 patients from a prospective database (2014–2018) who underwent PD; one hundred and eighty‐three patients were excluded because they had less than 1‐year of follow‐up, tumor recurrence during postoperative year one, or cholangitis from anastomotic stenosis or tumor recurrence. RNOC was defined as at least 3 episodes of cholangitis that occurred or persisted more than 1‐year after PD, without anastomotic stenosis or tumor recurrence. RF were identified by univariate and multivariate analyses.

**Results:**

Of the 320 analyzed patients, 213 (67%) and 107 (33%) underwent PD for malignancy and a benign lesion, respectively. With a median follow‐up of 47 months (IQR: 33–68), 27 of the 320 patients (8%) developed RNOC (median number of episodes = 8 and IQR = 5–12). The only independent RF for RNOC was noninvasive IPMN (OR = 3.222; IC‐95% = 1.219–8.514; and *p* = 0.018). All patients received on‐demand antibiotics. Three patients (11%) developed complications from RNOC (hepatic abscesses in 3 and organ failure in one). No patients died from RNOC. Two (7%) patients underwent reoperation to lengthen the biliary Roux‐en‐Y loop but RNOC persisted in both.

**Conclusions:**

RNOC complicates 8% of PD. Noninvasive IPMN was a RF of RNOC. The value of revision surgery to limit the risk of recurrence seems limited.

**Abbreviations**
CBD: common bile duct
DGE: delayed gastric emptying
HJ: hepaticojejunostomy
IPMN: intraductal papillary mucinous neoplasm
IQR: interquartile range
MV: multivariate
PD: pancreaticoduodenectomy
POPF: postoperative pancreatic *fistula*
PPH: postpancreatectomy hemorrhage
RF: risk factor
RNOC: recurrent nonobstructive cholangitis

## 1 Introduction

Pancreaticoduodenectomy (PD) is mainly indicated as a curative treatment for cancer [1]. Due to advances in perioperative management, postoperative mortality following PD is now less than 4% but morbidity remains at nearly 50% [1, 2, 3]. The indications for PD have been extended to premalignant or benign tumors [4]. Moreover, long‐term survival of patients with pancreatic adenocarcinoma has improved [5]. Thus, postoperative follow‐up is longer and previously rare complications are more frequent [6]. Indeed, most studies reporting morbidity following PD have focused on short‐term outcome [7, 8, 9].

Cholangitis occurs in the first month after PD in 5%–10% of patients [10, 11] or later with a prevalence of 6%–19% [6, 12, 13, 14]. Cholangitis is called “recurrent” or “refractory” after 2 or 3 episodes, whatever the cause and the postoperative delay [12, 15]. Etiological diagnosis of cholangitis should differentiate obstruction from benign stenosis or tumor recurrence [12, 14, 15, 16] from a nonobstructive mechanism [12, 16].

Recurrent nonobstructive cholangitis (RNOC) has been described not only after bilioenteric anastomosis for benign diseases [10, 16, 17] but also after PD when no anatomical biliary obstruction is present [14]. RNOC is probably explained by intestinal reflux into the intrahepatic ducts through the hepaticojejunostomy (HJ) [12, 16]. This case–control study aimed to investigate the prevalence, the RF, and the management of RNOC following PD.

## 2 Methods

### 2.1 Patients and Surgical Technique

From 2014 to 2018, 503 patients underwent PD with Child reconstruction, including pancreaticojejunostomy then HJ 15–20 cm downstream on the first jejunal loop positioned through the mesocolon, and antecolic or retrocolic gastrojejunostomy (or duodenojejunostomy after pylorus preservation) performed 50 cm downstream on the same loop. No biliary stent was used.

### 2.2 Postoperative Course and Follow‐Up

Complications included any adverse events within 90 days after PD. Postoperative pancreatic fistula (POPF), delayed gastric emptying (DGE), and postpancreatectomy hemorrhage were diagnosed according to the International Study Group on Pancreatic Surgery [7, 8, 9]. Bile leakage was diagnosed according to the International Study Group on Liver Surgery [18]. Complications were graded according to the Clavien–Dindo classification [19]. In patients with cancer, a clinico‐biological evaluation and CT were performed every 3 months for the 2 first postoperative years then every 6 months. Patients with benign disease were evaluated every 6–12 months. CT or MRI was performed periodically in every patient and in any patient developing symptoms of cholangitis or biliary obstruction.

Data were extracted from a prospective database. Follow‐up was obtained from the electronic hospital files, or direct information from the patient, his/her gastroenterologist, or the general practitioner. Follow‐up ended in December 2022. This single‐center retrospective case–control study was performed according to the Declaration of Helsinki and approved by the ethics committee of the French Infectious Diseases Society (CER‐MIT, IRB no. 00011642). Informed patient consent was obtained from all participants.

### 2.3 Definition of Cholangitis and Recurrent Nonobstructive Cholangitis

Acute cholangitis was diagnosed according to modified 2018 Tokyo Guidelines [20], which classify diagnostic items into 3 categories: systemic inflammation (A), cholestasis (B), and imaging (C). Each category has 2 items: category A, « fever and/or shaking/chills » and « laboratory data: evidence of inflammatory response » and category B, « jaundice » and « laboratory data: abnormal liver function ». However, regarding category C, neither biliary dilatation nor cause of obstruction was observed in patients with RNOC, but early inhomogeneous parenchymal enhancement was observed at dynamic CT performed during recurrent attacks [21]. To confirm a diagnosis of acute cholangitis, one item was required from each of the three categories.

Recurrent cholangitis was defined as at least 3 episodes. Biliary or first jejunal loop obstruction was routinely screened by CT or MRI in patients with cholangitis. An upper GI contrast study and hepatobiliary iminodiacetic acid (HIDA) scan were performed in certain patients to detect enterobiliary reflux or abnormal emptying of the jejunal loop, respectively. In the event of biliary dilatation without tumor recurrence, benign fibrotic anastomotic stenosis and/or intrahepatic lithiasis was considered. Patients with recurrent cholangitis due to obstruction were excluded. When neither dilatation nor tumor recurrence was present, a diagnosis of nonobstructive cholangitis was considered.

Recurrent nonobstructive cholangitis (RNOC) was defined by at least 3 episodes of cholangitis occurring or persisting after postoperative year one, thus allowing to not consider patients with cholangitis in the only early postoperative period or during adjuvant treatment. Nonobstructive cholangitis were treated according to the French Society of Infectious Diseases Guidelines [22]. The clinical and microbiological characteristics of some patients have been previously reported [23].

### 2.4 Statistical Analysis

Patient characteristics were compared between those who did and did not develop RNOC. Quantitative variables were described using medians (interquartile range = IQR). Qualitative variables were described using numbers and analyzed with the chi‐squared or Fisher's exact tests depending on the number of each variable. Multivariate (MV) analysis of factors associated with RNOC was performed by logistic regression. Variables included in the MV were selected based on their clinical relevance. All statistical analyses were performed with the SAS V9.4 software (SAS Institute, NC, Cary) and *p* < 0.05 was considered to be statistically significant.

## 3 Results

### 3.1 Patients, Intraoperative Data, and Early Postoperative Course

**FIGURE 1:**
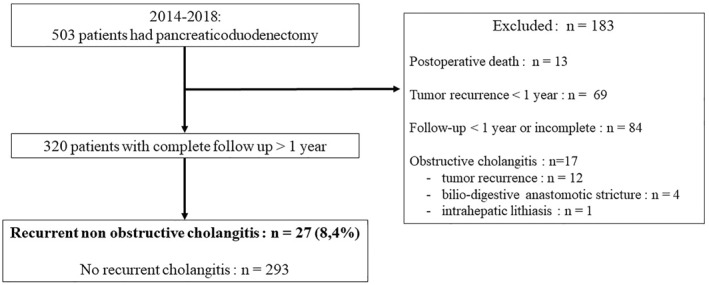
Study flow diagram.

Of the 503 patients who had PD, 183 (36.4%) were excluded from analysis due to early postoperative death (*n* = 13, 2.6%), tumor recurrence within one year (*n* = 69, 13.7%), follow‐up of less than one year or incomplete (*n* = 84, 16.7%), or obstructive cholangitis (*n* = 17, 3.4%). Of these 17 patients, 12 had tumor recurrence (diagnosed after 2 and 3 episodes in 9 and 3, respectively), 4 had benign anastomotic stenosis (diagnosed after 2 and 3 episodes in 2 and 2, respectively), and one had intrahepatic stones (Figure 1).

Finally, 320 patients were analyzed (Table 1). Median age was 61 (41–72) years. At presentation, diabetes and jaundice were present in 57 (17.8%) and 117 (36.6%), respectively. Neoadjuvant chemotherapy was given in 62 (19.4%) and neoadjuvant radiotherapy in 45 (14.0%). Preoperatively, 124 (38.8%) underwent endoscopic biliary drainage and 117 (36.6%) experienced cholangitis. The most frequent indications were pancreatic adenocarcinoma (*n* = 130; 40.6%) and noninvasive IPMN (*n* = 63; 19.7%).

**TABLE 1:** Preoperative data and indication of pancreaticoduodenectomy.

Variables	Overall cohort *n* = 320	Patients with RNOC *n* = 27	Patients without RNOC *n* = 293	*p* value
Preoperative characteristics				
Age (year)	61 (41–72)	64 (38–74)	60 (42–72)	0.129
Male	177 (55.3%)	16 (59%)	161 (55%)	0.667
Diabetes	57 (17.8%)	1 (3.7%)	56 (19.1%)	0.078
Body mass index (kg/m^2^)^a^	24.1 (21.3–32.8)	24.2 (20.3–31.2)	24.1 (21.7–32.9)	0.149
Initial symptoms				
None	68 (21.3%)	9 (33.3%)	59 (20.1%)	0.108
Present	252 (78.8%)	18 (66.7)	234 (79.9%)	
Ductal dilatation				
Common bile duct	140 (43.8%)	10 (37.0%)	130 (44.4%)	0.464
Main pancreatic duct	159 (49.7%)	13 (48.2%)	146 (49.9%)	0.867
Preoperative biliary drainage	124 (38.8%)	6 (22.2%)	118 (40.3%)	0.065
Neoadjuvant chemotherapy	62 (19.4%)	2 (7.4%)	60 (20.5%)	0.119
Indication of PD				
Pancreatic adenocarcinoma^b^	130 (40.6%)	7 (25.9%)	123 (42.0%)	0.110
Noninvasive IPMN	63 (19.7%)	13 (48.1%)	50 (17.1%)	< 0.001
Ampulloma	39 (12.2%)	2 (7.4%)	37 (12.6%)	0.434
Neuroendocrine tumor	36 (11.3%)	1 (3.7%)	35 (11.9%)	0.223
Cholangiocarcinoma	15 (4.7%)	1 (3.7%)	14 (4.8%)	0.801
Solid pseudopapillary tumor	9 (2.8%)	0 (0%)	9 (3.1%)	0.979
Duodenal adenocarcinoma	8 (2.5%)	2 (7.4%)	6 (2.0%)	0.111
Chronic pancreatitis	6 (1.9%)	1 (3.7%)	5 (1.7%)	0.475
Miscellaneous	0 (0%)	0 (0%)	14 (4.8%)	0.974
Malignancy	213 (66.6%)	12 (44.4%)	201 (68.6%)	0.011
Benign lesion	107 (33.4%)	15 (55.5%)	92 (31.4%)	

Abbreviations: IPMN, intraductal papillary mucinous neoplasm; PD, pancreaticoduodenectomy; RNOC, recurrent nonobstructive cholangitis.

^a^ Median values (range).

^b^ including invasive IPMN.

Intraoperatively (Table 2), the median CBD diameter was 8 (IQR: 6–12) mm. HJ was performed using monofilament absorbable running sutures in 181 (56.6%) patients or interrupted stitches in 139 (43.4%), respectively. The gastrojejunostomy (or duodenojejunostomy) was antecolic in 169 (52.8%) patients. Clavien–Dindo III–IV morbidity occurred in 52 (16.3%) patients. Grade B–C POPF and all grades DGE occurred in 83 (25.9%) and 89 (27.8%) patients, respectively. Early biliary complications occurred in 20 (6.3%) including biliary fistula in 11 (3.4%) and cholangitis in 9 (2.8%). No patient received adjuvant radiotherapy. Median follow‐up was 47 [IQR: 33–68] months, including 50 [IQR: 35–72] and 45 [IQR: 34–66] months in patients with and without RNOC, respectively (*p* = 0.07).

**TABLE 2:** Intraoperative data and day‐90 postoperative complications.

	Overall cohort *n* = 320	Patients with RNOC *n* = 27	Patients without RNOC *n* = 293	*p* value
Intraoperative data				
Laparotomy (vs. laparoscopy)	281 (87.8%)	21 (77.7%)	260 (88.8%)	0.132
Pylorus preservation	62 (19.4%)	6 (22.2%)	56 (19.1%)	0.673
Venous resection	54 (16.9%)	3 (11.1%)	51 (17.4%)	0.408
Pancreas consistency				
Hard	179 (55.9%)	13 (48.2%)	166 (56.7%)	0.396
Soft	141 (44.1%)	14 (51.8%)	127 (43.3%)	
Main pancreatic duct diameter (mm)^a^	4 (2–5)	4 (2–6)	4 (2–5)	0.963
Common bile duct diameter (mm)^a^	8 (6–12)	7 (5–11)	9 (6–12)	0.111
Biliary anastomosis				
Interrupted stitches	139 (43.4%)	15 (55.5%)	124 (42.3%)	0.183
Running suture	181 (56.6%)	12 (44.4%)	169 (57.7%)	
Digestive reconstruction				
Antecolic	166 (51.9%)	12 (44.4%)	154 (52.6%)	0.946
Retrocolic	154 (48.2%)	15 (55.5%)	139 (47.4%)	
Operative time (min)^a^	255 (230–420)	260 (220–350)	250 (230–420)	0.730
Estimated blood loss (mL)^a^	330 (300–700)	320 (250–800)	330 (300–750)	0.842
Postoperative course				
Morbidity according to Clavien–Dindo				0.699
0	86 (26.8%)	5 (18.5%)	81 (27.6%)	
I–II	182 (56.9%)	16 (59.3%)	166 (56.7%)	
IIIa–IIIb	27 (8.4%)	3 (11.1%)	24 (8.2%)	
IVa–IVb	25 (7.8%)	3 (11.1%)	22 (7.5%)	
Biliary fistula	11 (3.4%)	2 (7.4%)	9 (3.1%)	0.235
Cholangitis	9 (2.8%)	2 (7.4%)	7 (2.4%)	0.189
Delayed gastric emptying grade A/B/C	89 (27.8%)	10 (37.0%)	79 (27.0%)	0.267
Grade B/C postoperative pancreas fistula	83 (25.9%)	11 (40.7%)	72 (24.6%)	0.210
Surgical reoperation	20 (6.3%)	3 (11.1%)	17 (5.8%)	0.284
Arterial embolization	18 (5.6%)	2 (7.4%)	16 (5.5%)	0.675

Abbreviation: RNOC, recurrent nonobstructive cholangitis.

^a^ Median values (range).

## 4 Patients With RNOC

During or after postoperative year one, 27 (8.4%) patients developed RNOC. The presentation of the 3 initial episodes included: fever ≥ 38.5°C (*n* = 24, 88.9%), pain (*n* = 18, 66.7%), chills (*n* = 7, 25.9%), and jaundice (*n* = 2, 7.4%). All 27 patients had a biological inflammatory syndrome (hyperleukocytosis and/or elevated C‐reactive protein) with a transient increase in transaminases and alkaline phosphatases in 22 (81.5%) and 24 (88.9%) patients, respectively, whereas serum bilirubin level was ≤ 20 micromol/L in 22 (81.4%). The median number of episodes was 8 (IQR = 5–12) distributed as follows: 3–5: *n* = 11, 6–9: *n* = 8, and 10 or more: *n* = 8. Blood cultures were performed for at least one of the 3 initial episodes of RNOC in all 27 patients and were positive in 18 patients (66.7%). The three most frequent germs were as follows: *Escherichia coli* (*n* = 12, 44.4%), *Klebsiella* sp. (*n* = 4, 14.8%), and *Enterococcus* sp. (*n* = 3, 11.1%). Between two episodes, liver function tests were normal in 21 (77.8%) patients whereas 4 (14.8%) had persistent mild (< 1.2 fold the upper normal level) increase in alkaline phosphatase levels.

### 4.1 Risk Factors for RNOC

In univariate analysis, no statistically significant difference regarding presentation were noted between 27 patients with RNOC and 293 patients without RNOC (Table 1). There was a trend toward less diabetes (3.7% vs. 19.1%) and biliary drainage (22.2% vs. 40.3%) in the RNOC group. The prevalence of noninvasive IPMN was higher in the RNOC group: 13 (48.1%) versus 50 (17.1%) and *p* < 0.001. Overall, a benign lesion was more frequent in the RNOC group: 15 (55.5%) versus. 92 (31.4%) and *p* = 0.01. Intraoperative data were comparable between groups (Table 2). Regarding early biliary complications and POPF, there was no difference between patients with or without RNOC. To identify RF of RNOC, factors included in MV analysis were age, preoperative biliary drainage, neoadjuvant treatment, CBD diameter, noninvasive IPMN, and POPF. In MV analysis, noninvasive IPMN was the only independent RF associated with RNOC (OR = 3.222; IC‐95% = 1.219–8.514; and *p* = 0.018) (Table 3).

**TABLE 3:** Risk factors of recurrent nonobstructive cholangitis: multivariate analysis.

Characteristics	Odds ratio	95% CI	*p*
Age	1.026	[0.985–1.069]	0.225
Preoperative biliary drainage	0.915	[0.277–3.027]	0.889
Neoadjuvant treatment	0.660	[0.136–3.210]	0.607
CBD diameter	0.947	[0.813–1.103]	0.483
Noninvasive IPMN	3.222	[1.219–8.514]	0.018
Grade B–C POPF	1.590	[0.673–3.759]	0.291

Abbreviations: 95%‐CI, 95% confidence interval; CBD, common bile duct; IPMN, intraductal papillary mucinous neoplasm; POPF, postoperative pancreatic fistula.

## 5 Treatment and Prognosis of RNOC

All patients received intravenous antibiotics for the initial episodes of RNOC, whenever possible after blood cultures. A beta‐lactamine was given in 20 (74%) patients (amoxicillin/clavulanic acid, piperacillin/tazobactam, or cefepim combined with metronidazole) for 7 (IQR: 5–8) days. For well‐tolerated episodes of RNOC, oral antibiotics were prescribed. Duration of antibiotics was reduced to 5 days and then 3 days after 2018 according to recent guidelines. In patients with more than 3 episodes, the most recent ones were treated by empirical oral antibiotics without new blood cultures. Antibiotics effectively treated the symptoms in all patients except 3 (11.1%), whose antibiotics were modified after several episodes due to germ resistance. No patients died from RNOC but 3 (11.1%) developed complications including hepatic abscesses in 3 and hemodynamic failure in one (3.7%). Overall, the most severe episode was graded 1, 2, and 3 in 17, 9, and one patients, respectively.

**FIGURE 2:**
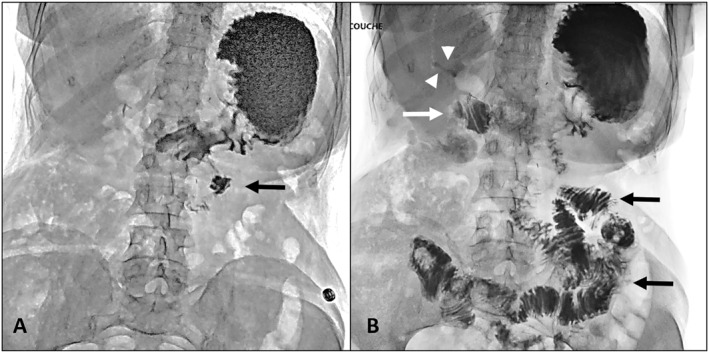
Upper GI contrast meal showing contrast reflux into the biliary tree after pancreaticoduodenectomy. (A) Early phase showing contrast medium in the stomach and the proximal efferent loop (black arrow) and (B) 10 min later, contrast medium can be seen in the afferent loop (white arrow) and in the right hepatic duct (white arrowheads).

Sixteen patients received a prolonged treatment by ursodesoxycholic acid, but only 2 (13%) described a mild decrease in the frequency of cholangitis episodes. PPI discontinuation or anti‐inflammatory drugs were not tested. Four patients received periodic courses of antibiotics, resulting in a decreased frequency of episodes in 2 (50%). Jejunal loop dysfunction was suspected in 2 patients after assessment with CT, MRI, upper GI contrast (Figure 2), and HIDA scan, who were reoperated to build a specific 70 cm Roux‐en‐Y jejunal loop anastomosed to the CBD. No anomaly was noted intraoperatively and surgery failed with persisting RNOC in both patients. Finally, one improved with somatostatin analogs and the other one was diagnosed with liver hamartomatosis.

## 6 Discussion

In the present series, RNOC was defined at least 3 episodes of cholangitis occurring or persisting after postoperative year one and occurred in 8.4% of our cohort of 320 patients after excluding tumor recurrence and obstructive cholangitis. Noninvasive IPMN was the only identified RF of RNOC with a prevalence up to 20% (13/63) in this entity and 14% (15/107) in benign diseases. Antibiotics were usually effective in almost all episodes but did not prevent recurrence. Periodic courses of antibiotics to decrease the risk of recurrence were given in four patients and were partially effective in two. Revision surgery to lengthen the jejunal loop anastomosed to the CBD was performed in two patients with disappointing results. These results suggest that RNOC is a fairly common complication of PD, especially when indicated for benign disease, and that it should be considered a significant long‐term drawback.

Most reports of cholangitis following PD have included all etiologies. In one study [24], the prevalence of late cholangitis was 16% with fibrous anastomotic stenosis and tumor recurrence as causes of cholangitis in 21% and 36% of patients, respectively, whereas 43% had no identified cause. These latter patients (7% of the entire series) might have developed nonobstructive cholangitis. In a second study [25], 28 (21%) patients had at least one (maximum: 5) episode of cholangitis; the first one occurred within the first year in 54%, within 2 years in 82% and after 3 years in 18%. The cause was anastomotic stenosis in 25% and possibly reflux in 39% but this series did not identify the cases of cholangitis due to tumor recurrence. In a third study [12], “refractory” cholangitis (at least 3 episodes) occurred in 19% of patients and was due to biliary strictures in half of them suggesting that the remaining half had RNOC. Fukuoka et al. used internal stents during HJ with a higher incidence of cholangitis, due to lack of stent migration [15]. Lastly, cholangitis following PD was reported in 10% of patients with at least two episodes in 47%; biliary obstruction was identified in 40%; and nonobstructive cholangitis in 60% [14]. Overall, very few studies have defined precisely nonobstructive cholangitis or used the number of episodes to define “recurrent” disease to avoid misdiagnosis with obstruction [15, 26]. Indeed, a diagnosis of benign HJ stenosis is difficult as reported after surgical biliary repair [27]. Thus, in the present study, to determine precisely the actual incidence of RNOC, we defined enterobiliary reflux as the cause of RNOC after at least 3 episodes with repeated imaging showing neither stenosis nor tumor recurrence, resulting in a rate of 8.4%. Regarding presentation of RNOC, we observed jaundice in only 2 (7.4%) patients and pain was absent in 9 (33%). The frequent absence of both jaundice and pain and the rarity of complications (11.1% in the present series and none reported in the series by Henry et al. [14]) suggest that nonobstructive cholangitis is less severe than obstructive cholangitis [14, 23, 26]. Contrast‐enhanced CT is not only useful for diagnosis for RNOC to exclude obstruction or tumor recurrence but also to visualize inhomogeneous enhancement at the early phase, which is present in up to 85% of patients with cholangitis [21].

The etiology of RNOC remains unclear. Theoretically, the reflux of food debris into the biliary tree is favored by a too short jejunal loop between the HJ and the gastrojejunostomy, or by a loop compression in the mesocolon window, or by bowel dysmotility or delayed gastric emptying. In the present study, we used a standard reconstruction technique including a 50 cm jejunal loop between HJ and gastrojejunostomy [13, 28]. Compression of the jejunal loop by the mesocolon seems unlikely since, to our knowledge, it has been never been reported except with tumor recurrence and was not present in our two reoperated patients. We also observed that postoperative DGE was not associated with a higher risk of RNOC but DGE completely resolve over time [9]. Bowel dysmotility likely explains RNOC since it is a mechanism of recurrent cholangitis following Roux‐en‐Y HJ in certain patients [29] or of fishbone migration into the intrahepatic bile ducts after PD [30] as well as a cause of biliary reflux after total gastrectomy or antireflux surgery [31]. Bowel dysmotility could explain: (i) the similar rates of cholangitis after choledocoduodenostomy and HJ for cholelithiasis in a randomized trial [17]; (ii) constipation as a possible RF of cholangitis following PD [15]; and (iii) the inefficiency of revision surgery creating a specific 70 cm Roux‐en‐Y loop in our two patients including one finally improving with somatostatin analogs.

The present study identified noninvasive IPMN as an independent RF of RNOC. A systematic review previously identified benign disease as RF of cholangitis following PD [32]. In a series of 900 PD, the only independent RF was biliary leakage but both obstructive and nonobstructive cholangitis were merged, whereas in the patients with nonobstructive cholangitis, male sex was the only independent RF [14]. In the present series, the high prevalence (33%) of benign diseases, mainly noninvasive IPMN and a longer postoperative follow‐up, could explain the 8.4% rate of RNOC.

Treatment of RNOC is mainly based on antibiotics. Theoretically, every episode should be documented by a blood culture and treated with antibiotics based on the most frequent germs (*E. coli*, *Klebsiella* sp, and *Enterococcus* spp) then possibly modified according to the antibiogram. For nonobstructive cholangitis, French [22] and other guidelines [33] recommend a short course of antibiotics for a duration recently reduced from 5 to 3 days. A short course is supported by the rarity of organ failure in RNOC: 3.7% in the present series and none in a series of 56 patients [14] and in another one of 19 patients [26]. Also, empirical antibiotics can be prescribed in patients who already experienced RNOC, after informing them about starting their treatment early after symptoms. The present study tested periodic discontinuous antibiotics to prevent recurrence in 4 patients, but only 2 patients experienced a decrease in the frequency of episodes; due to this low efficiency and the risk of selection of resistant germs, it cannot be recommended. We also tested ursodesoxycholic acid with poor results. We did not evaluated withdrawal of PPI, which are very frequently given following PD to limit the risk or marginal ulceration [34], but is suspected to favor the risk of cholangitis [35].

Revision surgery for RNOC has a limited role. Two of our patients with suspected jejunal dysfunction underwent revision surgery to redo HJ with no further improvement. However, successful revision has been reported to lengthen the jejunal loop after PD [36] or to switch an Imanaga to a child reconstruction [37]. In another study [14], two patients with RNOC after PD underwent reoperation to create a Roux‐en‐Y loop anastomosed to CBD with only one success. Lastly, a systematic review identified 9 cases of revision surgery to suppress RNOC after PD or other procedures including an HJ, with a 56% success rate [38]. Thus, revision surgery for RNOC should be indicated cautiously after a thorough work‐up evaluating the initial reconstruction and eliminating other causes of cholangitis [39].

The best reconstruction technique limiting the risk of RNOC after PD remains unknown. Bilioenterostomy per se is probably associated with a 10%–15% risk of nonobstructive cholangitis as observed after both choledocoduodenostomy and Roux‐en–Y HJ for CBD stones [17] or Roux‐en–Y HJ for benign CBD stenosis [10, 16]. As reported by high‐volume centers, we routinely performed a Child reconstruction with 50 cm of jejunum between the HJ and the gastrojejunostomy [28]. To our knowledge, only one study preferred a 60 cm loop [16]. Interestingly, neither pylorus preservation nor the gastro (duodeno)‐jejunostomy position influenced the rate of RNOC in the present study.

Our study has several limitations, including a high rate of PD for benign disease particularly noninvasive IPMN, the retrospective collection of data for patients treated for RNOC outside our institution, a certain number patients with short follow‐up, and heterogeneous work‐up to exclude obstruction.

In conclusion, RNOC is observed in 8% of patients following PD but more frequently after PD for noninvasive IPMN. Each episode of RNOC should be treated by a short course of antibiotics and, in well‐informed patients, by oral antibiotics without admission. Secondary prevention by ursodesoxycholic acid, discontinuous antibiotherapy, or revision surgery is debatable. Patients should be informed preoperatively of the risk of postoperative RNOC, particularly before PD for benign disease.

## Author Contributions

**Anaïs Choquet:** data curation, investigation, writing – original draft, writing – review and editing. **Aurélien Sokal:** data curation, investigation, writing – original draft, writing – review and editing. **Safi Dokmak:** data curation, formal analysis, writing – original draft, writing – review and editing. **Audrey Le Bot:** data curation, formal analysis, investigation, writing – original draft, writing – review and editing. **Fanny Delahaye:** formal analysis, methodology, writing – original draft, writing – review and editing. **Jeanne Dembinski:** formal analysis, methodology, software, writing – original draft, writing – review and editing. **Béatrice Aussilhou:** data curation, formal analysis, writing – original draft, writing – review and editing. **Vinciane Rebours:** data curation, supervision, validation, writing – original draft, writing – review and editing. **Victoire de Lastours:** conceptualization, investigation, methodology, supervision, writing – original draft, writing – review and editing. **Alain Sauvanet:** conceptualization, formal analysis, supervision, validation, writing – original draft, writing – review and editing.

## Conflicts of Interest

The authors declare no conflicts of interest.
